# Mentalizing Subtypes in Eating Disorders: A Latent Profile Analysis

**DOI:** 10.3389/fpsyg.2020.564291

**Published:** 2020-11-30

**Authors:** Giulia Gagliardini, Salvatore Gullo, Valeria Tinozzi, Monica Baiano, Matteo Balestrieri, Patrizia Todisco, Tiziana Schirone, Antonello Colli

**Affiliations:** ^1^Department of Humanities, “Carlo Bo” University of Urbino, Urbino, Italy; ^2^Department of Psychology, Educational Science and Human Movement, University of Palermo, Palermo, Italy; ^3^Centre for Eating Disorders, Azienda Sanitaria Universitaria Friuli Centrale (ASUFC), Udine, Italy; ^4^Psychiatric Clinic, Department of Medicine, University of Udine, Udine, Italy; ^5^Unit for Eating Disorders, Villa Margherita, Arcugnano, Italy

**Keywords:** mentalization, eating disorders, reflective functioning, anorexia, bulimia

## Abstract

**Background:** Mentalizing, the mental capacity to understand oneself and others in terms of mental states, has been found to be reduced in several mental disorders. Some studies have suggested that eating disorders (EDs) may also be associated with impairments in mentalizing. The aim of this work is to investigate the possible presence of mentalizing subtypes in a sample of patients with EDs.

**Method:** A sample of patients with eating disorders (*N* = 157) completed a battery of measures assessing mentalization and related variables, including the Reflective Functioning Questionnaire (RFQ), the Difficulties in Emotion Regulation Strategies (DERS), the Interpersonal Reactivity Index (IRI). Clinicians rated patients in relation to imbalances in different dimensions of mentalization to prementalizing modes and attachment style by using the Mentalization Imbalances Scale, the Modes of Mentalization Scale (MMS), and the Adult Attachment Questionnaire. A latent profile analysis was conducted to test the possible presence of different subgroups. MANOVA was used to test the possible differences between the four mentalizing profiles in relation to emotion dysregulation (DERS), empathy (IRI), and adequate and impairments in mentalizing (MMS and RFQ).

**Results:** The latent profile analysis suggested the presence of four different profiles in relation to impairments in the dimensions of mentalization: (1) affective/self/automatic imbalances, (2) external imbalance, (3) cognitive/self/automatic imbalances, and (4) cognitive/other/automatic imbalances. Patients belonging to profile 1 are characterized by the prevalence of affective mentalization that overwhelms the capacity to reflect on mental states with an imbalance on the self-dimension; profile 2 patients are excessively focused on the external cues of mentalization; profile 3 patients are characterized by an over-involvement on the cognitive and self-facets of mentalization, with an impairment in adopting the other mind perspective; and profile 4 patients have similar impairments compared to profile 3 patients but with an excessive focus on others and deficits in self-reflection. These profiles were heterogeneous in terms of EDs represented in each group and presented significant differences on various variables such as attachment style, emotion dysregulation, empathy, interpersonal reactivity, and reflective function. This study represents, so far, the first work that confirms the presence of different mentalizing patterns in ED patients.

**Conclusions:** ED patients can be classified in relation to impairments in different dimensions of mentalization above and beyond ED diagnosis.

## Introduction

(Tasca, 2019) in recent years, the concept of mentalization has attracted increasing research interest in the field of eating disorders (EDs), and several studies have reported findings of mentalizing difficulties in adults with EDs (Russell et al., 2009; Rothschild-Yakar et al., 2010; Oldershaw et al., 2011; Dejong et al., 2013; Morris et al., 2014). Moreover, some authors have suggested that ED symptomatology may be related to specific, peculiar difficulties in mentalization (Skårderud, 2007). Mentalization can be defined as “the mental process by which an individual implicitly and explicitly interprets the actions of himself and others as meaningful on the basis of intentional mental states such as personal desires, needs, feelings, beliefs, and reasons” (Bateman and Fonagy, 2004, p. 21) and is considered as a multifaceted ability related to eight different dimensions: automatic mentalization, controlled mentalization, mentalization toward self, mentalization toward others, cognitive mentalization, affective mentalization, internally focused mentalization, and externally focused mentalization (Luyten et al., 2012). The automatic dimension refers to the implicit and unconscious processes of recognizing and understanding inner mental states in oneself and others, while controlled mentalization refers to a conscious and deliberate action, such as when we talk about our feelings or motivations. The self and others dimensions are related to the object of mentalizing. Mentalization can, in fact, refer to the capacity to reflect on our own inner experiences, such as when we describe our emotions and/or feelings in a particular situation (the “mentalizing toward self” dimension), but it can also be focused on other people. The cognitive dimension of mentalization refers to the activity of understanding the representational nature of thoughts, while affective mentalization is a particular type of affective regulation that is composed of three different domains (Jurist, 2005): identifying, processing, and expressing affective mental states. Mentalization can be focused either on the external manifestations of mental states (e.g., prosody, body posture, face expressions, etc.) or on the inner world of individuals (e.g., beliefs, desires, etc.).

The literature on EDs and mentalization has theoretically suggested the presence of specific failures of mentalization in these patients (Skårderud, 2007). From an empirical standpoint, different authors have enlightened the presence of mentalizing problems in patients with ED (see, e.g., Aloi et al., 2017; Maxwell et al., 2017; Simonsen et al., 2020). A previous study on 25 patients with EDs (*n* = 13 with anorexia-restricting type, *n* = 7 with anorexia binge/purge type, and *n* = 5 with bulimia nervosa) has suggested how these patients are characterized by lower levels of mentalization regarding the self and higher levels of alexithymia, using more emotional suppression and less cognitive reappraisal than controls (Rothschild-Yakar et al., 2018). Several studies have examined the mentalizing ability with the Reflective Functioning (RF) Scale (Fonagy et al., 1998) and have shown that patients with anorexia nervosa (AN) exhibit a less developed ability to mentalize compared to individuals without EDs (Ward et al., 2001; Rothschild-Yakar et al., 2018). The ability to understand mental states in these patients seems to be related to emotional mental states and not to un-emotional mental states (Brockmeyer et al., 2016). In a previous study, Aloi et al. (2017) found that patients with binge eating disorder showed a deficit in recognizing their own emotions, with higher levels of alexithymia and problematics in interoceptive awareness.

Moreover, patients with AN have problems in relation to the ability to self mentalize and present imbalances on the cognitive dimension of mentalization as expressed by a high level of alexithymia compared with patients with bulimia nervosa (BN) and controls (Corcos et al., 2000; Speranza et al., 2005). Furthermore, a recent meta-analysis has reported that emotion recognition in others is impaired both in AN and BN patients. However, more severe deficits were found in acute AN, while BN was associated with only a small impairment (Bora and Köse, 2016).

A recent review and meta-analysis has suggested that patients with ED may show specific problematics in different dimensions of mentalization, with more severe difficulties on the self dimension of mentalization (Simonsen et al., 2020). The results of this analysis are consistent with the study of Adenzato et al. (2012), who found that patients with AN scored significantly lower on mentalization ability about themselves than healthy controls but exhibited a mentalization ability about others comparable with the healthy subjects. Aloi et al. (2017) likewise found that patients with binge eating disorders scored significantly lower on mentalization ability about themselves than healthy subjects but showed a comparable mentalizing ability about others.

In another study on a sample of 53 patients with BN with borderline features compared to a healthy control group, the authors found that patients with BN had significantly different scores compared to healthy controls on all tests of mentalizing. More specifically, BN patients showed significantly lower levels on RFQc (excessive certainty) and significantly higher levels on RFQu (excessive uncertainty), compared to healthy controls, on the Reflective Functioning Questionnaire (RFQ, see measures section), with moderate to large between-group effect sizes. These differences were related both to bulimic symptoms and to borderline personality disorder (BPD) features, suggesting that poor mentalizing may be a significant factor in BN patients and should be addressed in treatment, regardless of the presence of BPD features (Sacchetti et al., 2019). On the contrary, other studies have failed to find mentalizing impairments in BN (Pedersen et al., 2012), and some studies have shown that bulimic patients tend to have average or even high mentalizing abilities (Kenyon et al., 2012; Pedersen et al., 2015).

A study on 70 patients with BN has shown a bi-modal distribution of RF in these patients: The authors have hypothesized that these patients may be divided in two groups, one being more defended against mental states (with low RF scores) and one being more focused on the effort to understand others' behaviors (with higher RF scores) (Pedersen et al., 2012). On the whole, the abovementioned studies seem to suggest that ED patients show a specific pattern of problematics in emotion recognition and in mentalizing toward self, with some patients showing even high levels of mentalization.

The results of the studies mentioned so far seem to suggest that ED patients have problematics in mentalization but that these problematics are quite heterogeneous or not coherent across different studies. This partial incoherence in the results could be explained in relation to the different measures used in the various studies: for example, the RFS (Fonagy et al., 1998) represents a multidimensional measure of mentalization which provides only a global score ranging from −1 to +9 on the basis of a semi-structured interview focused on the patients' attachment relationships. This method has been used in previous studies (see, e.g., Taubner et al., 2013); however, a single global score does not provide information on the specific failures of mentalization and thus fails to encompass all the nuanced facets of mentalization which have been developed and studied over time. Therefore, while the RFS may not be so adequate at distinguishing good mentalization and hypermentalization, the RFQ seems to be more sensitive to these differences. Moreover, in the aforementioned studies, the rating of the different dimensions of mentalization (self, other, cognitive, affective, internal, external, implicit, explicit) or of the prementalizing modalities of thought described in the theoretical literature (pseudomentalization, concrete comprehension, teleological thought, good mentalization) was accomplished by using indirect methods (e.g., by using measures for the assessment of alexithymia to rate the self and affective dimension of mentalization) and not with *ad hoc* measures.

During the past few decades, the vast majority of research on mental illness has focused on investigating manifestations and correlates of categorical psychiatric diagnoses as defined by the DSM-5 (American Psychiatric Association, 2013). During recent years, however, concerns have been raised that categorical diagnoses are heterogeneous (i.e., subsuming clinically relevant subgroups of patients; Lilienfeld and Treadway, 2016). In the case of EDs, for example, several reviews have demonstrated that symptom presentations can vary to a relatively large extent within the diagnostic categories (Wildes and Marcus, 2015). Additionally, it has been shown that individuals cross over and shift between ED diagnoses over time, and some authors suggested that there may be common denominators, such as emotion dysregulation (Eddy et al., 2008; Lavender et al., 2015) or mentalization (Vann et al., 2014). The attempt to classify ED patients above and beyond symptoms and categorical diagnosis is not new, but these attempts focused generally on personality styles (Westen and Harnden-Fischer, 2001) or according to their evolution within the framework of care (Montourcy et al., 2018) rather than on specific factors that could be implicated in the etiology and maintenance of the disorders: e.g., previous research on the relationship between EDs and personality disorders has identified three personality subtypes in patients with EDs (Westen and Harnden-Fischer, 2001): a dysregulated/undercontrolled pattern, characterized by emotional dysregulation and impulsivity; a constricted/overcontrolled pattern, characterized by emotional inhibition, cognitively sparse representations of the self and others, and interpersonal avoidance; and a high-functioning/perfectionistic pattern, characterized by psychological strengths alloyed with perfectionism and negative affectivity.

The first aim of this work was to investigate the characteristics of mentalization in a sample of patients with EDs in order to identify different groups characterized by specific impairments in mentalization, independent from the ED symptoms. The second aim of this work was to investigate the relationship between these empirically derived mentalizing profiles and clinical, personality, and ED variables. In doing so, we focused on specific variables which have been previously found to be compromised in EDs and which are theoretically and empirically related to mentalization, i.e., personality pathology, attachment style, emotion dysregulation, cognitive and affective empathy interpersonal abilities, and reflective function. These variables are differently related to specific facets of mentalization which, in previous studies, were found to be compromised in patients with EDs, more specifically the recognition of emotions and the understanding of others and own's mental states. This is an exploratory study which aimed at filling the literature gap on the presence of specific mentalizing profiles in ED patients. Given the exploratory nature of this study and since the available literature on the topic does not allow for specific conclusions and points out the necessity to have further studies before being able to identify specific mentalizing problematics in ED patients (Simonsen et al., 2020), no specific hypotheses on the profiles were made.

## Materials and Methods

### Sampling Procedure

From the rosters of the major societies of psychodynamic and cognitive–behavioral psychotherapy and from centers that specialized in the treatment of eating disorders, we contacted 700 psychotherapists and asked for their willingness to participate in the study. We requested that they select a patient who was at least 18 years old, had had no psychotic disorder or psychotic symptoms for at least the last 6 months, had seen the therapist for a minimum of eight sessions and a maximum of 18 months, and had an ED diagnosis. Following the same procedure adopted in similar studies (Betan et al., 2005; Colli et al., 2014), we asked clinicians to rate each randomly ordered criterion for each of the DSM-5 ED diagnoses (American Psychiatric Association, 2013) as present or absent. This procedure provided both a categorical diagnosis (by applying DSM-5 cutoffs) and a dimensional measure (number of criteria met for each disorder). To minimize selection biases, we directed the clinicians to consult their calendars to select the last patient they had seen during the previous week who met the study criteria. To minimize rater-dependent biases, each clinician was allowed to describe only one patient. We contacted 700 clinicians, of whom 157 returned their measures, for an overall response rate of 22.5%. The clinicians received no remuneration. The final sample is composed of 157 therapeutic dyads. The present study was approved by the Institution Review Board (Ethics Committee) of the “Carlo Bo” University of Urbino (Italy).

### Patients

The sample was composed of 157 Caucasian patients with ED, treated in psychotherapy. One hundred forty-nine (94.9 %) were female and eight (5.1%) were male (mean age = 30.88; SD = 11.95; min. = 18; max. = 65). The patients were diagnosed with different EDs, more specifically: AN (*n* = 64, 41.4%; of which 44 with AN restricting type and 20 with AN binge purge type), BN (*n* = 41, 26.3%), binge eating disorder (BED) (*n* = 27, 16.2%), other specified feeding or eating disorder (*n* = 13, 8.4%), and unspecified feeding or eating disorder (*n* = 12, 7.7%). Sixty-nine patients (44%) had at least one previous hospitalization. Seventeen patients (10.8%) had attempted suicide at least once, and 33 were reported with self-harming behaviors (21%). Eighty-nine (57.8%) were, by the time of the assessment, undergoing a pharmacotherapy. Fifty-one patients also had a personality disorder (PD) diagnosis according to the DSM-5 (American Psychiatric Association, 2013), alone or in comorbidity, and fifty-seven patients had sub-threshold personality problems. The average length of treatment at the moment of the evaluation was 10.31 months (SD = 12.13; min. = 3; max. = 70).

### Therapists

The sample was composed of 157 Caucasian therapists, of which 116 (73.9%) were female and 41 (26.1%) were male (mean age = 43.21; SD = 8.61; min. = 30; max. = 65). Four theoretical and clinical approaches were represented in this sample: psychodynamic (*n* = 61), systemic (*n* = 33), integrative (*n* = 30), cognitive (*n* = 19), and other approaches (e.g., humanistic, bioenergetic, interpersonal; *n* = 14). The clinicians had an average of 15.22 years of previous clinical experience as psychotherapists (SD = 8.07; min. = 2; max. = 35). Thirty-five therapists (22.3%) were seeing the selected patients in public health services, 31 (19.8%) in hospitals, 30 (19.1%) a private setting, 22 (14%) in residential structures, and 35 in different settings (schools, universities, forensic, *etc*.).

### Measures/Instruments

The therapists were asked to fill out different measures. The clinician report measures included:

- Mentalization Imbalances Scale (MIS) (Gagliardini et al., 2018): The MIS represents a clinician report assessment measure of mentalizing imbalances in adult patients. It is composed of 22 items rated on a Likert scale from 0 (“absolutely not descriptive”) to 5 (“absolutely descriptive”) and represents an assessment measure of mentalizing imbalances on the basis of six subscales: imbalance toward self (four items), indicating an excessive focus on patient's own mind which prevents from the possibility to connect with others' thoughts and feelings and perspectives; imbalance toward others (three items), indicating an excessive focus on other peoples' mental states rather than the patient's own; affective imbalance (four items), indicating a hyper-activation of affects and emotions not adequately balanced by cognition; cognitive imbalance (five items), indicating an excessive focus on the cognitive facets of mentalization (which can lead to intellectualizing) that is not balanced by the affective facets of experience; automatic imbalance (three items), indicating the ability to automatically and implicitly recognize mental states, which, however, is not paired by the capacity to explicitly and declaratively reflect on them, even when actively solicited by others (e.g., a therapist); and external imbalance (three items), indicating those cases in which a person excessively relies on the external cues of mental states (i.e., facial expressions, body postures, *etc*.) without reflecting on inner mental states (e.g., beliefs, desires, thoughts, emotions). The MIS has been used in previous studies (Carrera et al., 2018; Gagliardini et al., 2018, 2020) in which it has shown a good reliability. In the present study, the scale has shown sufficient to good psychometric properties (Cronbach, 1951), with alphas of 0.84 (imbalance on the self), 0.80 (cognitive imbalance), 0.79 (automatic imbalance), 0.78 (affective imbalance), 0.61 (imbalance on the others), and 0.60 (external imbalance).
- Modes of Mentalization Scale (MMS) (Gagliardini and Colli, 2019): The MMS is a clinician report assessment measure of the modes of mentalization on five different subscales: (1) excessive certainty (six items), indicating an over-activation of mentalization, in which patients show an excessive certainty about mental states and think that they can provide all of the answers regarding other people's inner worlds; (2) concrete thinking (six items), indicating the tendency to interpret reality on the basis of heuristics and prejudices and/or on the basis of physical or invariant constraints, to use common sense explanations or clichés to explain emotions, and to adopt bizarre explanations of behaviors; (3) good mentalization (five items), indicating a good capacity to recognize and coherently describe mental states, united with a curious stance toward the same and an awareness that people can experience contrasting feelings and desires; (4) teleological thought (three items), indicating a tendency to rely more on the physical manifestations of mental states (i.e., actions) rather than interpreting the world in terms of beliefs, desires, or thoughts, to focus more on what people do (and not on what they think or feel), and to be more focused on the physical, practical, resolution of a problem rather than on the meanings related to the situation; (5) intrusive pseudomentalization (four items), related to a more malign form of hyper- or pseudo-mentalization, indicating a tendency to intrude on and manipulate other people's life, in which the reflections of one's inner world do not seem to be genuine. The factor structure and reliability of the scale was explored in previous studies (Gagliardini and Colli, 2019; Gagliardini et al., 2020) that enlightened good psychometric properties, with alphas ranging from 0.91 to 0.67 (Gagliardini and Colli, 2019). In the present study, alphas were ranging from sufficient to good (Cronbach, 1951) and were, respectively, 0.88 (excessive certainty), 0.86 (good mentalization), 0.83 (concrete comprehension), 0.75 (teleological thought), and 0.71 (intrusive pseudomentalization).
- Eating disorders: Following the same procedure adopted in similar studies (Betan et al., 2005; Colli et al., 2014), we asked the clinicians to rate each randomly ordered criterion for each of the DSM-5 ED diagnoses (American Psychiatric Association, 2013) as present or absent. This procedure provided both a categorical diagnosis (by applying DSM-5 cutoffs) and a dimensional measure (number of criteria met for each disorder).
- Clinical questionnaire: The clinical questionnaire was constructed *ad hoc* for clinicians in order to obtain general information about them, their patients, and the therapies they used. Clinicians provided basic demographic and professional data, including discipline (psychiatry or psychology), theoretical approach, hours of work, and gender as well as patients' ages and other concomitant therapies (e.g., pharmacotherapy). Clinicians provided additional data on the therapies, such as length of treatment and number of sessions per week. To provide a more comprehensive assessment of patients' problems that may be connected to EDs and/or to mentalizing deficits, the respondents were also asked to use the items of the clinical questionnaire to rate the presence or the absence of a list of clinical problems (American Psychiatric Association, 2013), such as dissociative symptoms, self-harming behaviors, and obsessive symptoms.
- Personality disorders checklist: Following the same procedure adopted in similar studies (Betan et al., 2005; Colli et al., 2014), we asked the clinicians to rate each randomly ordered criterion for each of the DSM-5 PD diagnoses (American Psychiatric Association, 2013) as present or absent. This procedure provided both a categorical diagnosis (by applying DSM-5 cutoffs) and a dimensional measure (number of criteria met for each disorder).
- Adult Attachment Questionnaire (AAQ) (Westen and Nakash, 2006): The AAQ is a 37-item clinician report measure designed to assess patients' attachment styles. It is based on a seven-point Likert scale and codifies patients' attachment styles into four different dimensions: secure (“tends to expect that s/he can rely on the availability and responsiveness of the people who are important to him/her”), insecure-dismissing (“tends to minimize or dismiss the importance of close relationship with others”), insecure-preoccupied [“seems to be mired in, or preoccupied with, past attachment relationships (*e.g*., seems still to be fighting old battles with mother, father, *etc*.)”] and incoherent/disorganized [“tends to use vague, meaningless, or empty words when describing interpersonal events (*e.g*., may insert nonsense words such as ‘dadadada’ into sentences or use psychobabble such as ‘she has a lot of material around that issue’)”]. The AAQ has been used in previous studies on Italian samples, in which it showed good psychometric properties (see, *e.g*., Gagliardini and Colli, 2019). In the present study, alphas were, respectively,0.85 (secure),0.83 (insecure-preoccupied),0.80 (disorganized), and.69 (insecure-dismissing).

The patients' self-report measures included:

- Difficulties in Emotion Regulation Strategies (DERS) (Gratz and Roemer, 2004): The DERS is a self-report measure filled out by patients and is composed of six subscales for the assessment of emotional regulation: (1) lack of acceptance of the emotional responses (“non-acceptance”), (2) difficulty in distracting from emotions and engaging in goal-oriented behaviors (“goals”), (3) limited access to emotion regulation strategies (“strategies”), (4) lack of control when experiencing intense emotions (“impulse”), (5) difficulties in recognizing emotions (“clarity”), and (6) limited awareness of emotion (“awareness”) (Weinberg and Klonsky, 2009; Neumann et al., 2010; Perez et al., 2012; Ritschel et al., 2015). In this study, we adopted the Italian version of the scale, which has been validated by Sighinolfi et al. (2010). In the present study, the scale has shown good psychometric properties (Cronbach, 1951), with alphas ranging from 0.91 to 0.83.
- Basic Empathy Scale (BES) (Jolliffe and Farrington, 2006; Albiero et al., 2009): The BES is composed of 20 items assessed on a Likert scale from 1 (“strongly disagree”) to 5 (“strongly agree”). The scale provides a measure for empathic concern on two dimensions: affective empathy and cognitive empathy. The scale has shown a good internal consistency and reliability in relation to different measures for the assessment of related constructs (Carré et al., 2013). Empathy, as assessed with the BES, correlates with intelligence, extroversion, and neuroticism. The Italian validation of the BES (Albiero et al., 2009) has also indicated a positive correlation between the BES and interpersonal reactivity as assessed with the Interpersonal Reactivity Index (Davis, 1980); moreover, higher empathy scores are related to prosocial behaviors. In this study the scale has shown good psychometric properties, with alphas of 0.82 (affective empathy) and of 0.83 (cognitive empathy).
- Interpersonal Reactivity Index (IRI) (Davis, 1980; Albiero et al., 2006): The IRI represents a measure for the assessment of empathic responsiveness and is composed of 28 items rated by patients on a five-point Likert scale. It is composed of four subscales: (1) fantasy, which assesses the tendency to transpose one's self imaginatively into the feelings and actions of fictitious characters; (2) empathic concern, covering feelings for others such as sympathy and concern for their welfare; (3) perspective taking, which describes one's tendency to spontaneously adopt the psychological point of view of others; and (4) personal distress, which is related to feelings of distress, unpleasantness, and anxiety resulting from tense interpersonal situations. The factor structure of the scale has been confirmed in different studies (Chrysikou and Thompson, 2016). In this study, the scale has shown good psychometric properties, with alphas of 0.83 (perspective taking), 0.75 (personal distress), 0.72 (empathic concern), and 0.71 (fantasy).
- Reflective Functioning Questionnaire (RFQ) (Fonagy et al., 2016; Morandotti et al., 2018): The RFQ is a self-report for the assessment of mentalization. The RFQ assesses mentalization or reflective function by asking the patient to answer eight items on a Likert scale from 1 (“strongly disagree”) to 7 (“strongly agree”). Scores are then recoded and collapsed into two different subscales: RFQ_certainty (RFQ_c), which reflects an excessive certainty about mental states, and RFQ_uncertainty (RFQ_u), which reflects an excessive uncertainty about self and others' mental states. The factor structure of the scale has been tested on a sample of patients with eating disorders and borderline PD (Fonagy et al., 2016). In this study, the scale has shown sufficient psychometric properties, with alphas of 0.65 (RFQ_u) and 0.70 (RFQ_c), respectively.

### Statistical Analysis

To empirically discriminate the presence of specific profiles in relation to mentalizing impairments in patients with Eds, we performed a latent profile analysis (LPA) according to their scores on the six subscales of the Mentalization Imbalances Scale. Previously to this analysis, outliers and univariate normality were checked. The following criteria were used for deciding the best number of profiles to be retained: non-significant *p*-values for the bootstrapped likelihood ratio test (BLRT), lower values of Akaike Information Criterion (AIC) and of Bayesian Information Criterion (BIC), and entropy value higher than 0.80 (Wang et al., 2017). MANOVA and ANOVA were used to detect differences among the empirically determined profiles in the self-report measures. Zero-order correlations were calculated to test associations between study variables and profile posterior probabilities. Chi-square test was used to investigate differences in the distribution of ED diagnosis among the profiles. LPA was conducted through the R package “TidyLPA”; all other analyses were performed using SPSS Statistics 21 for Windows (IBM, Armonk, NY, USA).

## Results

### Descriptive Statistics

Table 1 shows the descriptive statistics for MIS, MMS, BES, DERS, IRI, and RFQ in the whole sample (*N* = 157). Preliminary analyses showed that none of the MIS subscales contained outliers. Further data inspection showed no violations of normality for all the subscales (standardized |skewness| and |kurtosis| <1.0), with the exception of the BES cognitive empathy scale (kurtosis = 2.32). Table 1 shows the means and standard deviations for MIS, MMS, BES, DERS, IRI, and RFQ in the whole sample (*N* = 157).

**Table 1:** Descriptive statistics for the MIS, MMS, AAQ, DERS, BES, RFQ, and IRI (*N* = 157).

**MIS**	***M***	**SD**
Cognitive imbalance	2.32	1.08
External imbalance	2.24	1.06
Affective imbalance	2.59	1.28
Imbalance toward others	2.40	1.16
Imbalance toward self	2.28	1.22
Automatic imbalance	2.28	1.22
**MMS**		
Excessive certainty	1.59	1.05
Concrete comprehension	1.76	1.01
Good mentalization	2.80	1.10
Teleological thought	2.50	1.19
Intrusive pseudomentalization	1.25	1.00
**AAQ**		
Secure	3.14	1.01
Insecure dismissing	2.41	0.92
Insecure preoccupied	2.74	1.21
Disorganized	1.87	1.03
**DERS**		
Non-acceptance	3.07	1.13
Goals	3.58	1.00
Strategies	3.19	0.83
Impulse	2.87	1.04
Clarity	3.11	0.94
Awareness	2.65	1.10
**BES**		
Affective empathy	3.68	0.69
Cognitive empathy	3.85	0.52
**RFQ**		
Uncertainty	5.88	3.86
Certainty	5.29	3.64
**IRI**		
Fantasy	3.05	0.75
Empathic concern	3.79	0.69
Perspective taking	3.30	0.73
Personal distress	3.15	0.70

*MIS, Mentalization Imbalances Scale; MMS, Modes of Mentalization Scale; AAQ, Adult Attachment Questionnaire; DERS, Difficulties in Emotion Regulation Scale; BES, Basic Empathy Scale; IRI, Interpersonal Reactivity Index; RFQ, Reflective Functioning Questionnaire*.

### Latent Profile Analysis

**Figure 1:**
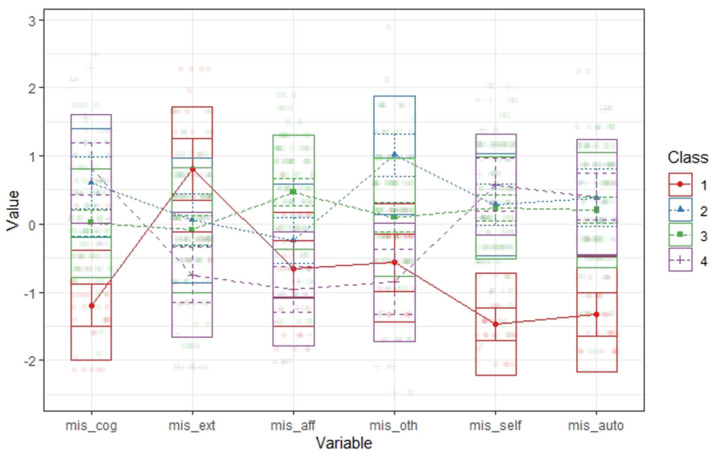
Standardized group averages on MIS subscales for a four-profile solution. MIS, Mentalization Imbalances Scale; cog, cognitive imbalance; ext, external imbalance; aff, affective imbalance; oth, imbalance toward others; self, imbalance toward self; auto, automatic imbalance; E, external; CAO, cognitive automatic other; CSA, self-cognitive automatic; ASA, affective self-automatic.

To identify the optimal number of profiles, models with one through five profiles (*k* = 1–5) were compared. Fit statistics, including the BLRT, AIC, BIC, and entropy values are presented in Table 2. An analytic hierarchy process, based on these fit indices (Akogul and Erisoglu, 2017), suggested that the best solution was the model with four profiles. The comparisons between overall mean for each scale score of mentalization and within-profile mean were used to assign labels to the four empirically derived profiles (Table 3). Figure 1 presents the standardized group averages on MIS subscales for a four-profile solution.

**Table 2:** Fit statistics of the latent profile analysis (*k* = 1:5).

**Number of profiles (*k*)**	**BLRT**	**AIC**	**BIC**	**Entropy**
1	/	2,503.95	2,598.29	
2	81.48^*^	2,470.96	2,640.40	0.61
3	77.13^*^	2,434.71	2,631.25	0.80
4	51.20	2,378.34	2,622.97	0.86
5	46.95	2,450.65	2,646.38	0.92

* *p < 0.05*.

*BLRT, bootstrapped likelihood ratio test; AIC, Akaike Information Criterion; BIC, Bayesian Information Criterion*.

**Table 3:** Comparison between overall mean and within-profile means.

	**Overall mean**	**“E” profile (*****N*** **=** **26)**		**“CAO” profile (*****N*** **=** **24)**		**“ASA” profile (*****N*** **=** **87)**		**“CAS” profile (*****N*** **=** **20)**	
		**Mean difference**	***t***	**Mean difference**	***t***	**Mean difference**	***t***	**Mean difference**	***t***
MIS cognitive	2.32	−1.30	−8.35^*^	*0.62*	3.78^*^	0.01	0.06	*0.90*	5.00^*^
MIS extern	2.24	*0.86*	3.89^*^	0.08	0.43	−0.09	−0.89	−0.84	−4.54^*^
MIS affective	2.59	−0.92	4.63^*^	−0.29	−1.44^*^	*0.63*	*5.36*	−1.21	−6.26^*^
MIS other	2.16	−0.55	−5.42^*^	*0.87*	7.20^*^	0.09	1.05	−0.74	−4.27^*^
MIS self	2.40	−1.78	−24.62^*^	0.29	1.91	*0.28*	2.76^*^	*0.73*	3.57^*^
MIS auto	2.28	−1.70	−17.53^*^	*0.43*	2.17^*^	*0.27*	2.42^*^	*0.49*	2.45^*^

* *p < 0.05*.

*MIS, Mentalization Imbalances Scale; cognitive, cognitive imbalance; extern, external imbalance; affective, affective imbalance; other, imbalance toward others; self, imbalance toward self; auto, automatic imbalance; E, external; CAO, cognitive automatic other; CSA, cognitive self-automatic; ASA, affective self-automatic. MIS, Mentalization Imbalances Scale; E, External; CAO, Cognitive Automatic Other; CSA, Cognitive Automatic Self; ASA, Affective Self-Automatic*.

The proportions of subjects within each profile showed that more than half of patients (55%) had higher-than-average score on affective, self, and auto imbalances; in this group, patients' emotions overcome his/her capacity to think, and they may feel that emotions are out of control (ASA). The second most numerous profile included 17% of patients and was focused on external imbalance (E); in this profile, the patient tends to focus more than sample's average on others' facial expressions and/or non-verbal cues when communicating with other; he/she may easily be influenced by other peoples' emotions. The last two profiles shared high scores on cognitive and automatic scales, but their imbalances were oriented toward different objects being focused to understand their own (CSA; 12%) or other people's (COA; 15%) mental states more on a cognitive level than on an affective one and throughout implicit and uncontrolled processes. Table 4 presents a narrative prototype based on the most descriptive MIS items for each profile.

**Table 4:** Narrative prototypes based on the most descriptive MIS items for each profile.

**Profile 1 “E”**
The patient seems to preverbally intuit people's feelings or thoughts (3.29) and is excessively focused on others' facial expressions and/or non-verbal cues when communicating with others (including the therapist) (3.06). The patient seems to have a “sixth sense” about other people's (including the therapist) mental states (2.77). Moreover, the patients' emotions seem to overcome their capacity to think (2.69), and they may easily be influenced by other peoples' emotions (2.64). The patient feels that emotions are out of control (2.33) and seems to be unconsciously attuned to other people's emotions (2.23).
**Profile 2 “CAO”**
The patient seems to understand people more on a cognitive level than on an affective one (3.60), and when speaking, he/she seems to be detached from emotions (3.17). The patient often seems to lack words to describe feelings (3.11), and they may easily be influenced by other peoples' emotions (3.11). Moreover, the patient seems to inhibit the expression of (positive and/or negative) emotions (3.06) and seems to be unconsciously attuned to other people's emotions (2.97).
**Profile 3 “ASA”**
The patients' emotions overcome his/her capacity to think (3.78), and they may feel that emotions are out of control (3.69). In turn, the patients may act impulsively (3.52) and may misinterpret other people's behavior (3.45). The patients can easily be influenced by others' emotions (3.34) but may have problems at understanding others' perspective when interpreting their behaviors (3.33). The patients' emotions can change rapidly (3.29).
**Profile 4 “CSA”**
The patient seems to understand people more on a cognitive level than on an affective one (3.60), and when speaking, he/she seems to be detached from emotions (3.17). The patient often seems to lack words to describe feelings (3.11) and fails to assume others' perspective when interpreting behaviors (3.11). Moreover, the patient seems to inhibit the expression of (positive and/or negative) emotions (3.06). The patient fails to consider points of view that differ from his/her own (2.97) and may misunderstand other people's behaviors (2.94).

*MIS, Mentalization Imbalances Scale; E, external; CAO, cognitive automatic other; CSA, cognitive self-automatic; ASA, affective self-automatic*.

The four profiles identified were heterogeneous in terms of ED distribution (see Table 5). We found no statistical difference in relation to eating disorders' diagnoses among the profiles, *X*^2^ (*df* = 15) = 24.10, *p* = 0.06.

**Table 5:** ED and PD distributions among the four profiles.

	**“E” profile** **(*N* = 26)**		**“CAO” profile** **(*N* = 24)**		**“ASA” profile** **(*N* = 87)**		**“CSA” profile** **(*N* = 20)**	
	***n***	**%**	***n***	**%**	***n***	**%**	***n***	**%**
AN (restrictive)	5	19.2	13	56.5	18	21.2	8	40.0
AN (binge/purge)	2	7.7	2	8.7	13	15.3	3	15.0
BN	9	34.6	1	4.3	26	30.6	4	20.0
BED	7	26.9	2	8.7	3	15.3	3	15.0
Other specified ED	1	3.8	4	17.4	7	8.2	1	5.0
Not specified ED	2	7.7	1	4.3	8	9.4	1	5.0
PD	2	1.3	10	7.2	34	22	8	5.1

*AN, anorexia nervosa; BN, bulimia nervosa; BED, binge eating disorder; ED, eating disorder; PD, personality disorders*.

### Profiles and Other Variables

In order to give a deeper description and characterization of profiles and testing their clinical utility, we were interested in comparing the four profiles in relation to different facets of mentalization such as prementalizing modes (Modes of Mentalization Scale), certainty/uncertainty about mental states (RFQ), and other variables related with mentalization such as attachment (AAQ), emotional regulation (DERS), empathy (BES), and interpersonal sensitivity.

Multivariate ANOVA was routinely used to compare the profiles in relation to other study variables (see Supplementary Table 1). However, because of the small sample size (*N* < 30) of three out of the four profiles that would have reduced the power of the study, we chose to treat the data as continuous variables. In fact, in the LPA, the assignment of the participants to the classes is based on their posterior modal probabilities; in other terms, each subject has a membership probability value for each of the *k* profiles identified. Therefore, we calculate the correlations between the posterior probabilities for each profile and the above-mentioned study variables (Table 6).

**Table 6:** Correlations between mentalization profiles and prementalizing modes, attachment, emotion regulation, and empathy.

	**“E” profile**	**“CAO” profile**	**“ASA” profile**	**“CSA” profile**
**MMS**				
Excessive certainty	−0.271^***^	0.019	0.195^*^	−0.002
Concrete comprehension	−0.443^***^	0.157	0.271^***^	−0.064
Good mentalization	0.542^***^	−0.114	−0.218^**^	0.173^*^
Teleological thought	−0.445^***^	0.111	0.212^**^	0.073
Intrusive pseudomentalization	−0.359^***^	0.206^**^	0.157	−0.042
**AAQ**				
Secure	0.581^***^	−0.118^*^	−0.182^*^	−0.192^*^
Insecure dismissing	−0.397^***^	0.093	0.073	0.224^**^
Insecure preoccupied	−0.229^**^	−0.021	0.243^**^	−0.076
Disorganized	−0.339^***^	0.076	0.254^***^	−0.071
**DERS**				
Non–acceptance	−0.048	−0.229^**^	0.236^**^	−0.058
Goals	−0.057	−0.150	0.159	−0.016
Strategies	−0.161	−0.061	0.255^**^	−0.126
Impulse	−0.054	−0.046	0.294^***^	−0.319^***^
Clarity	−0.117	0.025	0.127	−0.077
Awareness	−0.169^*^	0.018	0.173^*^	−0.082
**RFQ**				
Uncertainty	−0.135	−0.091	0.250^**^	−0.118
Certainty	0.091	0.042	−0.174^*^	0.108
**BES**				
Affective empathy	0.093	−0.004	0.036	−0.155
Cognitive empathy	0.177^*^	−0.109	−0.032	−0.041
**IRI**				
Fantasy	0.171^*^	−0.137^**^	0.108	−0.108
Empathic concern	0.091	−0.071	0.173^*^	−0.280^***^
Perspective taking	0.194^*^	−0.203^*^	−0.023	0.023
Personal distress	−0.064	0.035	0.134	−0.158

* *p < 0.05*,

** *p < 0.01*,

*** *p < 0.001*.

*MIS, Mentalization Imbalances Scale; AAQ, Adult Attachment Questionnaire; MMS, Modes of Mentalization Scale; DERS, Difficulties in Emotion Regulation Scale; RFQ, Reflective Functioning Questionnaire; BES, Basic Empathy Scale; IRI, Interpersonal Reactivity Index*.

In relation to the MMS, higher probabilities of being in ASA profile result in being significantly associated with several prementalizing modes (concrete, teleological, and excessive certainty); the CAO profile results in being associated with intrusive pseudomentalization. Higher probabilities of belonging to the CSA and the ASA profile results in being negatively associated with good mentalization. Conversely higher membership or assignment probabilities in E profile result in being associated in a positive way with good mentalization and negatively with all prementalizing modes. Moreover, higher membership in ASA group was associated with higher RFQ uncertainty and lower RFQ certainty.

In relation to emotion regulation, higher membership in ASA profile results in being associated with several dimensions of DERS, confirming that this profile was composed of patients with greater difficulties in emotion regulation. Conversely, the two profiles characterized by imbalances on the cognitive dimension (CAO and CAS) higher membership result in being negatively associated with the non-acceptance and impulse DERS scale, respectively.

In relation to empathy (IRI), the perspective taking dimension results in being negatively associated with higher membership in CAO profile but positively with the external profile, while the empathic concern resulted higher when the probability of being in ASA profile was higher and lower for higher membership in CAS profile. Finally, the fantasy scale results in being associated with the E profile and negatively with the CAO. Concerning attachment styles, secure attachment results in being positively associated with higher membership in E profile and negatively associated with the other profiles, while preoccupied and disorganized attachment was positively associated with higher membership in ASA profile.

## Discussion

The aim of the present study was to investigate the possible presence of different groups of mentalization impairments in adult patients with EDs. Our results suggest the presence of four different profiles in relation to elevation in mentalization imbalances (MIS scores): external (E), cognitive-other-automatic (CAO), affective-self-automatic (ASA), and cognitive-self-automatic (CAS). The four profiles were heterogeneous not only in terms of mentalizing impairments but also in terms of different clinical aspects. This result seems to confirm findings of previous studies which have enlightened how patients with EDs may be more clearly identified by using different variables other than eating problematics and symptoms, variables which are more closely related to personality or mental functioning and less related to weight attention on the body and attitude toward food.

The profile we labeled ASA (with higher imbalances in affective, self-oriented, and automatic facets) seems to collect ED patients characterized by greater problematics in emotional regulation that have difficulties in accepting and trusting their own feelings, as also confirmed by their low certainty about their own mental states. Patients of the ASA profile, rather than adopting high-functioning or controlled reflection, tend to shift to prementalizing modes of thought such as teleological and concrete comprehension. In other words, these patients tend to interpret reality in terms of physical reality rather than considering complex mental states. Moreover, the affective focus of these patients probably allow them to have “other oriented” feelings and sympathy, but this is not associated with an ability in perspective taking. These patients showed greater problematics in relation to attachment, especially in relation to preoccupied and disorganized attachment. Finally, into the ASA profile, it falls more than half of the patients in our sample. This data can partially be explained by the fact that this seems to be the profile that is characterized by the more compelling problematics in mentalization and our patients have shown high levels of comorbidity with PDs. Many of the patients of the ASA profile, in fact, have a comorbid PD. A different explanation could be related to the fact that 75 of the patients of our sample have problematics in the control of impulses and have a diagnosis of AN binge/purge type, BN, and BED, and these problematics may be related to the affective and automatic facets of mentalization. This data is also in line with previous works which have enlightened the presence of problematics in emotion regulation in ED patients (Monell et al., 2018; Prefit et al., 2019).

Two profiles had results characterized by a greater focus on the cognitive facets of mentalization but differed on the basis of the focus on the self (CSA) or the others (CAO). It is worth of note that anorexia was the prevalent diagnosis in both of these two profiles. This seems to support the picture of a “cold” anorexic patient, cognitively rigid and affectively constricted (Schmidt and Treasure, 2006). However, these imbalances were also present in bulimic or binger individuals, suggesting that poor mentalization should be considered as a transdiagnostic impairment. Regarding the interpersonal area of functioning, it is interesting to note that the focus on the self and on the cognitive dimension of the CSA patients seems to reduce their capacity to feel what the others are experiencing (a reduced empathic concern), and this is also confirmed by the significant association with the dismissing attachment style. Moreover, the CAO patients' profile results in being negatively associated with perspective taking: this result seems to confirm that, in these patients, the difficulty at mentalizing and the subsequent incapacity to fully differentiate self and others do not allow for them to feel the authentic capacity to connect with others empathetically. In line with this consideration, this profile was associated with intrusive preseudomentalization, in which the opaqueness of the other mind is not respected.

A separate discussion is related to the results on the external profile, which is characterized by subjects that excessively rely on the external signs of mental states (i.e., facial expressions, body postures, etc.) (Luyten and Fonagy, 2015). In our study, this profile results in being associated with secure attachment, good mentalization, and perspective taking: in other words, this profile seems to be associated with several signs of high functioning in patients. Bateman and Fonagy (2016) has recently recalled that, in many studies, poor facial emotion recognition and communication, increased facial avoidance, and reduced understanding of mental states were all associated with eating disorder pathology. Moreover, there is empirical evidence of impaired recognition of facial emotions in AN in comparison to healthy individuals (Kucharska-Pietura et al., 2004). Therefore, the preference of this group of patients on external cues could indicate a higher functioning in this profile. However, from our point of view, this does not mean that the patients in this group are totally free of any mentalizing impairment, but they somehow seem to compensate them. These patients could be excessively focused on the external facets of mentalization, perhaps in order to vigilate on the external environment and control it. This result may explain the contradictory results on the relationship between BN and mentalization: It is possible that there are different mentalizing styles in bulimic patients, with one group showing higher mentalizing functioning and another group being more dysregulated and impaired in terms of mentalization. Therefore, this result seems to confirm the presence of a group of patients with ED that is characterized both by a focus on the effort to understand others' behaviors and high RF scores (Pedersen et al., 2012).

A different explanation is related to the validity of the external scale of the MIS, which probably fails to distinguish between a pathological and excessive external focus on the external facets of mentalization and the capacity to understand others' mental states on the basis of external cues such as face expression. In a previous study (Gagliardini et al., 2018), the authors have suggested that two of the items of this subscale [i.e., “Patient seems to preverbally intuit people's feelings or thoughts” and “The patient seems to have a “sixth sense” about other people's (including the therapist) mental states”] could be interpreted by clinicians as positive capacities and therefore could indicate the adaptive capacity of patients to connect and comprehend on a procedural basis others' mental states. Moreover, this is the only MIS scale which has not an opposite dimension of the pole (e.g., cognitive vs. affective). While it is quite easy to think about an affective imbalance as associated to difficulties in the cognitive facets of mentalization and *vice versa*, the same is not true for the internal/external dimension. From a phenomenological and clinical perspective, in fact, we may conceptualize an imbalance on the external facet of mentalization (e.g., a patient who sees the therapist frowning and therefore hypothesizes that he or she is bored); it is more difficult to hypothesize that the patients overestimate the internal facets of mentalization without considering the external cues of mentalizing, and this is more true for cognitive processes. For example, whereas the differentiation internal/external seems easily applicable to emotions, which have both external expressions and internal feeling qualities, this does not seem to be the case with cognitions, which do not have bodily expressions to any similar degree (Liljenfors and Lundh, 2015). In the future, it will be necessary to address the issue of the validity of this subscale.

The four-profile solution found in our study seems to be consistent with the findings of previous studies that tried to categorize eating disorders in different personality subtypes (Westen and Harnden-Fischer, 2001; Thompson-Brenner and Westen, 2005): the ASA profile, characterized first of all by the affective imbalance and an impairment in controlled mentalization, seems close to the dysregulated/undercontrolled pattern described in literature; the COA and CSA profiles that are both characterized by an excessive focus on the cognitive facets of experience and a form of detachment from emotions seem to echo the constricted personality subtype, and finally the external profile seems similar to the high-functioning personality group.

More generally, an interesting result of this study regards the distribution of mentalization imbalances between the different diagnoses of ED. This result can lead to various speculations: one could explore the existence of ED subtypes with different mentalization characteristics/difficulties; mentalization problem heterogeneity and transdiagnostic occurrence may also mark different etiological pathways for eating psychopathology. In line with this, it would be interesting to explore how mentalization impairments in ED patients are associated with the outcome of the therapy.

This study has some limitations. First and foremost, the distribution of EDs in our sample is heterogeneous but not balanced (41% of our patents were diagnosed with AN, restrictive or binge/purge type). Future studies should involve more balanced samples in which each ED is equally represented. The ED diagnoses provided in this study rely on the clinical judgement of the therapists included in the study, of whom were asked to rate each DSM-5 (American Psychiatric Association, 2013) ED criterion as present or absent. This procedure, which allows for us to have both dimensional and categorical diagnoses for each ED, has a major limitation of not being based on a structured interview, which would have increased the accuracy of the ED diagnoses. Moreover, there is a high comorbidity of PDs in the sample, and this could impact on the generalizability of our results: for example, we could not exclude that the profiles we found were largely influenced by the patients' personality problematics. However, it is important to observe that comorbidity with personality disorders is quite frequent in the case of EDs and that the comorbidity we observed in our sample is similar to the ones described in literature (Godt, 2008; Martinussen et al., 2017). Similarly, several patients were under pharmacological therapy, and this could affect their mental functioning and the profiles that we observed. The Cronbach alpha of some scales was below 0.70, and so the reliability of some results could not be sufficient.

Finally, the age range of the sample is quite wide (min. = 18, max. = 68), and so is the chronicity of the disorder. Unfortunately, our sample size did not allow for us to perform different latent profile analyses in different age groups; however, future studies should consider this possibility in order to provide more detailed information on the possible presence of different clusters related to the chronicity of the disease.

Despite these limitations and the exploratory nature of our study taken together, these results seem to suggest that a comprehensive assessment of patients with EDs should also be focused on the patients' mentalizing impairments since patients with the same ED could be characterized by opposite patterns of mentalizing impairments: for example, patients with AN were characterized by imbalances on both the cognitive and the affective dimensions of mentalization. From our point of view, this could also change the focus of clinicians' interventions, which should be in one case the affective facets of mentalization and on the other the cognitive dimension, by promoting emotion regulation strategies. The four profiles that have been identified in this study must be further investigated, and future studies should also investigate the treatment outcome of therapeutic interventions of these profiles in order to see if different profiles are associated with different outcomes.

## Conclusions

ED patients can be classified in relation to impairments in different dimensions of mentalization above and beyond ED diagnosis. This preliminary investigation suggests to clinicians to take in consideration mentalization from a multidimensional approach when treating ED patients.

## Data Availability Statement

The raw data supporting the conclusions of this article will be made available by the authors, without undue reservation.

## Ethics Statement

The studies involving human participants were reviewed and approved by Institutional Review Board of the University of Urbino. The patients/participants provided their written informed consent to participate in this study.

## Author Contributions

GG and AC were the principal investigators of the study, planned the research, analyzed data and prepared the manuscript. SG helped with the manuscript revision and provided significant contributions to the methodological and statistical sections of the manuscript. VT collected data. MBai, MBal, and PT collected data and completed evaluations. TS helped in revising the manuscript. All authors contributed to the article and approved the submitted version.

## Conflict of Interest

The authors declare that the research was conducted in the absence of any commercial or financial relationships that could be construed as a potential conflict of interest.
