# Supplementary material for: Mentalizing Subtypes in Eating Disorders: A Latent Profile Analysis
Source: Front Psychol. 2020 Nov 30;11:564291. doi: 10.3389/fpsyg.2020.564291 (PMC7734030; doi:10.3389/fpsyg.2020.564291)
Supplement: Supplementary file 1 [file Table_1.DOCX]

Supplementary Table S1. .Within-profile mean and MANOVA post-hoc results.

|  | “E”  profile  (N=26)  Mean | “COA” profile  (N=24)  Mean | “ASA” profile  (N=86)  Mean | “CSA” profile  (N=20)  Mean |  | MANOVA |  | Cohen’s d |
| --- | --- | --- | --- | --- | --- | --- | --- | --- |

|  |  |  |  |  |  | F | | Post-hoc | |  | E  *vs*  COA | E  *vs*  ASA | E  *vs*  CSA | COA  *vs*  ASA | COA  *vs*  CSA | ASA  *vs*  CSA |
| --- | --- | --- | --- | --- | --- | --- | --- | --- | --- | --- | --- | --- | --- | --- | --- | --- |
| MMS |  |  |  |  |  |  |  | |  |  |  |  |  |  |  |  |
| Excessive Certainty | 0.95 | 1.65 | 1.75 | 1.64 |  | 4.15** |  | | E < ASA |  | -0.77 | -0.75 | -0.71 | -0.09 | 0.01 | 0.10 |
| Concrete Comprehension | 0.78 | 2.10 | 1.98 | 1.65 |  | 13.03** |  | | E < COA, ASA, CSA |  | -1.86 | -1.31 | -1.11 | 0.12 | 0.51 | 0.33 |
| Good Mentalization | 4.13 | 2.54 | 2.59 | 2.29 |  | 21.70** |  | | E > COA, ASA, CSA |  | 1.77 | 1.75 | 2.04 | -0.05 | 0.24 | 0.32 |
| Teleological Thought | 1.36 | 2.79 | 2.69 | 2.78 |  | 11.63** |  | | E < COA, ASA, CSA |  | -1.67 | -1.18 | -1.45 | 0.09 | 0.01 | -0.08 |
| Intrusive Pseudomentalization | 0.49 | 1.70 | 1.38 | 1.18 |  | 8.18** |  | | E < COA, ASA, |  | -1.57 | -0.95 | -0.97 | 0.32 | 0.56 | 0.20 |
| AAQ |  |  |  |  |  |  |  | |  |  |  |  |  |  |  |  |
| Secure | 4.46 | 2.74 | 2.99 | 2.62 |  | 28.16** |  | | E > COA, ASA, CSA |  | 2.01 | 1.78 | 2.32 | -0.30 | 0.15 | 0.45 |
| Insecure Dismissing | 1.62 | 2.59 | 2.48 | 2.97 |  | 11.37** |  | | E < COA, ASA, CSA |  | -1.46 | -1.06 | -1.65 | 0.13 | -0.43 | -0.54 |
| Insecure Preoccupied | 2.13 | 2.67 | 2.99 | 2.57 |  | 3.69* |  | | E < ASA |  | -0.51 | -0.72 | -0.40 | -0.26 | 0.08 | 0.38 |
| Disorganized | 1.06 | 2.02 | 2.11 | 1.69 |  | 8.28** |  | | E < COA, ASA |  | -1.08 | -1.09 | -0.84 | -0.09 | 0.34 | 0.42 |
| DERS |  |  |  |  |  |  |  | |  |  |  |  |  |  |  |  |
| Non Acceptance | 2.96 | 2.47 | 3.29 | 2.94 |  | 3.05* |  | | ASA > COA |  | .44 | -0.30 | 0.02 | -0.74 | -0.45 | 0.32 |
| Goals | 3.46 | 3.24 | 3.70 | 3.56 |  | 1.09 |  | |  |  | 0.21 | -0.25 | -0.10 | -0.46 | -0.29 | 0.14 |
| Strategies | 2.91 | 3.07 | 3.37 | 2.96 |  | 2.96* |  | |  |  | -0.20 | -0.58 | -0.07 | -0.38 | 0.13 | 0.51 |
| Impulse | 2.72 | 2.77 | 3.13 | 2.08 |  | 6.55** |  | | ASA > CSA |  | -0.45 | -0.40 | 0.70 | -0.36 | 0.79 | 1.13 |
| Clarity | 2.87 | 3.17 | 3.21 | 2.90 |  | 1.17 |  | |  |  | -0.04 | -0.36 | -0.02 | -0.04 | 0.28 | 0.32 |
| Awareness | 2.27 | 2.72 | 2.83 | 2.42 |  | 2.11 |  | |  |  | -0.35 | -0.51 | -0.14 | -0.10 | 0.29 | 0.37 |
| RFQ |  |  |  |  |  |  |  | |  |  |  |  |  |  |  |  |
| Uncertainty | 0.81 | 0.84 | 1.13 | 0.77 |  | 3.20* |  | |  |  | -0.06 | -0.50 | 0.07 | -0.44 | 0.12 | 0.54 |
| Certainty | 1.01 | 0.96 | 0.77 | 1.09 |  | 1.45 |  | |  |  | 0.05 | 0.34 | -0.10 | 0.26 | -0.15 | -0.46 |
| BES |  |  |  |  |  |  |  | |  |  |  |  |  |  |  |  |
| Affective Empathy | 3.81 | 3.69 | 3.69 | 3.43 |  | 1.20 |  | |  |  | 0.20 | 0.18 | 0.54 | 0.00 | 0.36 | 0.36 |
| Cognitive Empathy | 4.04 | 3.71 | 3.84 | 3.83 |  | 1.69 |  | |  |  | 0.72 | 0.43 | 0.38 | -0.27 | -0.19 | 0.02 |
| IRI |  |  |  |  |  |  |  | |  |  |  |  |  |  |  |  |
| Fantasy | 3.81 | 3.69 | 3.69 | 3.43 |  | 4.28** |  | | COA < E, ASA |  | 0.99 | 0.23 | 0.61 | -0.69 | -0.37 | 0.36 |
| Empathic Concern | 4.04 | 3.71 | 3.84 | 3.83 |  | 3.81* |  | | CSA < E, ASA |  | 0.35 | 0.09 | 0.86 | -0.26 | 0.51 | 0.78 |
| Perspective Taking | 3.81 | 3.69 | 3.69 | 3.43 |  | 3.82* |  | | COA < E |  | 0.94 | 0.61 | 0.37 | -0.40 | -0.49 | -0.18 |
| Personal Distress | 4.04 | 3.71 | 3.84 | 3.83 |  | 1.90 |  | |  |  | -0.23 | -0.28 | 0.29 | -0.05 | 0.47 | 0.55 |

Note. MIS:Mentalization Imbalances Scale. AAQ:Adult Attachment Questionnaire. MMS:Modes of Menetalization Scale. DERS:Difficulties in Emotion Regulation Scale. RFQ:Reflective Functioning Questionnaire. BES:Basic Empathy Scale. IRI:Interpersonal Reactivity Index; Cohen’s *d* of 0.20 = 'small' effect size, 0.50 = 'medium' effect size and 0.80 = 'large' effect size.
